# Cell-Specific Protective Signaling Induced by the Novel AT2R-Agonist NP-6A4 on Human Endothelial and Smooth Muscle Cells

**DOI:** 10.3389/fphar.2018.00928

**Published:** 2018-08-21

**Authors:** Ryan Toedebusch, Anthony Belenchia, Lakshmi Pulakat

**Affiliations:** ^1^Department of Medicine, University of Missouri, Columbia, MO, United States; ^2^Dalton Cardiovascular Research Center, University of Missouri, Columbia, MO, United States; ^3^Department of Nutrition and Exercise Physiology, University of Missouri, Columbia, MO, United States

**Keywords:** NP-6A4, AT2R, RTCA, nitric oxide, mitochondrial energetics, ROS, JNK

## Abstract

Cardiovascular disease incidence continues to rise and new treatment paradigms are warranted. We reported previously that activation of Angiotensin II receptor (encoded by the X-linked *Agtr2* gene) by a new peptide agonist, NP-6A4, was more effective in protecting mouse cardiomyocyte HL-1 cells and human coronary artery vascular smooth muscle cells (hCAVSMCs) from acute nutrient deficiency than other drugs tested. To elucidate further the protective effects of NP-6A4 in human cells, we studied the effects of NP-6A4 treatment on functions of human coronary artery endothelial cells (hCAECs), and hCAVSMCs. In hCAVSMCs, NP-6A4 (1 μM) increased *Agtr2* mRNA (sixfold, *p* < 0.05) after 12-h exposure, whereas in hCAECs, significant increase in *Agtr2* mRNA (hCAECs: eightfold) was observed after prolonged exposure. Interestingly, NP-6A4 treatment (1 μM, 12 h) increased AT2R protein levels in all human cells tested. Pre-treatment with AT2R-antagonist PD123319 (20 μM) and anti-AT2R siRNA (1 μM) suppressed this effect. Thus, NP-6A4 activates a positive feedback loop for AT2R expression and signaling in hCAVSMCs and hCAECs. NP-6A4 (1–20 μM) increased cell index (CI) of hCAVSMCs as determined by real time cell analyzer (RTCA), indicating that high concentrations of NP-6A4 were not cytotoxic for hCAVSMCs, rather promoting better cell attachment and growth. Seahorse Extracellular Flux Assay revealed that NP-6A4 (1 μM) treatment for 7 days increased whole cell-based mitochondrial parameters of hCAVSMCs, specifically maximal respiration (*p* < 0.05), spare respiratory capacity (*p* < 0.05) and ATP production (*p* < 0.05). NP-6A4 (1 μM; 7 days) also suppressed Reactive Oxygen Species (ROS) in hCAVSMCs. Exposure to Doxorubicin (DOXO) (1 μM) increased ROS in hCAVSMCs and this effect was suppressed by NP-6A4 (1 μM). In hCAECs grown in complete medium, NP-6A4 (1 μM) and Ang II (1 μM) exerted similar changes in CI. Additionally, NP-6A4 (5 μM: 12 h) increased expression of eNOS (sixfold, *p* < 0.05) and generation of nitric oxide (1.3-fold, *p* < 0.05) in hCAECs and pre-treatment with PD123319 (20 μM) suppressed this effect partially (65%). Finally, NP-6A4 decreased phosphorylation of Jun-N-terminal kinase, implicated in apoptosis of ECs in atherosclerotic sites. Taken together, NP-6A4, through its ability to increase AT2R expression and signaling, exerts different cell-specific protective effects in human VSMCs and ECs.

## Introduction

Cardiovascular disease (CVD) is the leading cause of death in both men and women worldwide (World Health Organization [WHO], 2017). The WHO estimated that 17.7 million people died from CVD, accounting for 31% of all global deaths in 2015 (World Health Organization [WHO], 2017). The renin-angiotensin system (RAS) and angiotensin II (Ang II) play a pivotal role in normal cardiovascular physiology, including regulating systemic blood pressure, renal blood flow, and glomerular filtration rate (Inagami, 1994; Gul et al., 2012; Borghi et al., 2015). Chronic pathologies such as hypertension, obesity, diabetes, and metabolic syndrome are accompanied by dysregulation of RAS, and subsequent increased signaling via the Ang II type 1 receptor (AT1R). AT1R activation plays a major role in pathophysiological effects, including; vasoconstriction, fibrosis, oxidative stress via increased ROS, inflammation, and pathological remodeling of cardiovascular, cerebrovascular and renal tissues (Sadoshima and Izumo, 1993; Widdop et al., 2003; Ferrario and Strawn, 2006). Consequently, drugs that inhibit Ang II synthesis or block AT1R-signaling are the standard of care for diseases where RAS over-activation is an underlying cause.

However, Ang II also binds and activates a second receptor, AT2R, encoded by the X-linked *Agtr2* gene. Like AT1R, AT2R is a G-protein coupled receptor; but shares only 34% homology with AT1R (Kambayashi et al., 1993; Mukoyama et al., 1993). AT2R expression, which is high in multiple tissues during fetal development, is reduced in adult tissues and primarily seen in renal, neurological and cardiovascular systems in adult rats (Wang et al., 1998; Miyata et al., 1999). An increase in AT2R expression is observed in response to injury and pathophysiological remodeling (Masaki et al., 1998; Akishita et al., 2000; Li et al., 2005; Altarche-Xifro et al., 2009; Curato et al., 2010) indicating a critical role for AT2R in tissue repair and regeneration. However, mechanisms underlying this effect are not fully understood.

AT2R inhibits AT1R-mediated increase in inositol triphosphate by interacting with the third intracellular loop of AT1R (Kumar et al., 2002; Xu et al., 2014), which in turn, leads to vasodilation, anti-fibrotic, anti-proliferative, and anti-inflammatory effects (Widdop et al., 2003; Jones et al., 2008; Ludwig et al., 2012). Transgenic overexpression of AT2R promotes cardiac repair after myocardial infarction in mice (Xu et al., 2014). Chronic activation of AT2R renders renal protection in diabetic rats (Ali et al., 2013; Xu et al., 2014), and neuro-protection in hypertensive rats (McCarthy et al., 2014). Increased AT2R expression is seen in the vasculature of female mice and heart tissues of female rats compared to their male counterparts and this sex difference in AT2R expression is implicated in increased cardiovascular protection in females (Okumura et al., 2005; Sampson et al., 2008; Lum-Naihe et al., 2017). It is accepted that many of the beneficial effects of AT1R blockers (ARBs) are due to increases in the amount of bioavailable Ang II, which binds to and activates AT2R receptors (Oishi et al., 2006).

Although ARBs are used widely in the treatment of CVD, meta-analyses of randomized clinical trials suggest that ARBs are not as effective as expected in preventing pathologic remodeling, fibrosis and cardiomyopathy (Axelsson et al., 2015, 2016). Despite the potential of AT2R to promote cardiovascular repair, to date there are no approved AT2R agonists to treat CVD or its co-morbidities. Compound 21, a non-peptide AT2R agonist, is an emerging drug for the treatment of idiopathic pulmonary fibrosis and has been shown to offer protection in various tissues including brain (McCarthy et al., 2014; Fouda et al., 2017), vasculature (Chow et al., 2016), kidney (Pandey and Gaikwad, 2017), and heart (Gao et al., 2014) in various rodent disease models. One major challenge in using AT2R agonists to treat CVD is the reduced expression of AT2R in adult tissues, particularly in males. Studies in rodent models have shown that transgenic overexpression of AT2R is beneficial in cardiac repair. However, there are no reports that suggest Compound 21 increases AT2R mRNA or protein expression, limiting its protective benefits.

One important beneficial effect of AT2R activation is mediated through endothelial nitric oxide synthase (eNOS) and subsequent increases in nitric oxide (NO) (Nguyen Dinh Cat et al., 2013; Padia and Carey, 2013). NO is responsible for numerous beneficial functions due to its actions as a potent vasodilator, pro-survival, anti-inflammatory, and antioxidant agent. Cardiac AT2R activation has been associated with NO-mediated downstream benefits (Zhu et al., 2010), including vasodilation (Yayama and Okamoto, 2008) and attenuation of fibrosis (Kurisu et al., 2003). Compound 21 increased NO within the renal interstitial fluid in a model of type 1 diabetes (Matavelli et al., 2015) and in human aortic endothelial cells (Peluso et al., 2018).

NP-6A4 is a patent-pending peptide agonist from Novopyxis Inc. (Boston, MA, United States) which has garnered FDA’s Orphan Drug designation for the treatment of pediatric cardiomyopathy. We reported previously that NP-6A4 could protect mouse HL-1 cardiomyocytes and human coronary artery vascular smooth muscle cells from acute nutrient deficiency (Mahmood and Pulakat, 2015). To elucidate further the translational potential of NP-6A4, we investigated signaling activated by NP-6A4 in human ECs from two different origins, coronary artery and umbilical vein and hCAVSMCs when grown in complete medium. We report for the first time that NP-6A4 increases *Agtr2* mRNA and AT2R protein in human vascular cells, albeit the effects of NP-6A4 are in a cell-specific manner. Additionally, we show that NP-6A4 activates protective signaling mechanisms in human ECs that may play a role in reducing atherosclerosis and promoting cardioprotective signaling.

## Materials and Methods

### Cell Culture and Reagents

All cells were grown at 37°C and 5% CO_2_. hCAVSMCs (GIBCO – Invitrogen Cell culture, Carlsbad, CA, United States) were cultured in Medium 231 supplemented with Smooth Muscle Growth Supplement (Life Technologies). hCAECs (Cell Applications, Inc.) were cultured in MesoEndo Cell Growth Medium (Cell Applications, Inc.). Human Umbilical Vein Endothelial Cells (hUVECs – GIBCO-Invitrogen Cell culture, Carlsbad, CA, United States) were cultured in Medium 200 supplemented with Low Serum Growth Supplement (Life Technologies). PD123319 (AT2R antagonist) and Losartan (AT1R antagonist) were purchased from Tocris Bioscience (Bristol, United Kingdom). NP-6A4 was a gift from Novopyxis Inc. (Boston, MA, United States). All experiments were performed on cells between passage number three and six. For collection, cells were rinsed twice with ice-cold PBS, scraped, centrifuged at 180 × *g*, and flash frozen with liquid nitrogen for subsequent assays.

### RNA Isolation for qRT-PCR and DNA Isolation

Total RNA was isolated using the Direct-zol MiniPrep kit (Zymo Research, United States) according to manufacturer’s specifications, and DNase treatment was carried out on the column before RNA elution. Using one microgram purified RNA, cDNA was reverse transcribed using the High-Capacity cDNA Reverse Transcription Kit (Thermo Fisher – Applied Biosystems). Predesigned and validated TaqMan probes (Agt2r Assay ID: Hs02621316_s1 and Agtr1 Assay ID: Hs05043708_s1) and 2X master mix were obtained from Thermo Fisher (Rockford, IL, United States). qRT-PCR was performed in triplicate using 200 ng cDNA. mRNA expression values were quantified by the 2^-ΔΔCt^ method, whereby ΔCT = 18S Ct – gene of interest Ct. DNA was isolated using a commercially available kit for assaying mitochondrial DNA copy number (Detroit R&D Inc., Detroit, MI, United States), and provided mitochondrial and nuclear primers were used to generate the ratio of mtDNA/nuclear DNA using the following formula: ΔCt1 = Ct (*mitochondrial control*) – Ct (*nucleus control*)

### siRNA Mediated Knockdown of AT2R

In order to use an additional measure to antagonize and knockdown AT2R receptor expression, we utilized AT2R-targeted siRNA. Briefly, AGTR2 Silencer Select Pre-designed siRNA (ID: s1184, Cat#: 4392420) and Silencer Select Negative Control #1 siRNA (Cat#: 4390843) were purchased from Ambion (Ambion, United States). Transfection was carried out in hCAECs using siPORT Amine transfection reagent (Invitrogen – Thermo Fisher Scientific). Briefly, cells were seeded in 24-wells plates (treated with 12.5 μg/ml bovine fibronectin in 0.02% gelatin solution), and 50 μl 1:1 solution (siPORT Amine + OPTI-MEM: 1 μM siRNA or siRNA-scramble + OPTI-MEM) was added to respective wells for 24 h. NP-6A4 was added immediately after transfection. After 24 h, cells were fixed in 4% PFA and subjected to immunofluorescence protocol described below.

### Immunofluorescence

Immunofluorescence was used to determine the changes in the expression of AT2R protein in hCAVSMC, hCAEC, and hUVEC in response to NP-6A4 treatment. All cells were grown on cover slips in 24-well plates treated with fibronectin in appropriate media. PD123319 was applied (20 μM) 20 min before adding NP-6A4 (1 μM). NP-6A4-containing medium was replenished twice during 12-h treatment, to ensure availability of the drug. After 12-h treatments, coverslips were washed with HBSS (Sigma), fixed with 4% paraformaldehyde for 15 min at room temperature, permeabilized with 0.5% Triton X-100, washed with HBSS buffer, and blocked with 1% bovine serum albumin (BSA) (Jackson ImmunoResearch), along with 10% goat serum (Sigma) in PBS-T (1 mL Tween-20/L). Coverslips were incubated with anti-AT2R antibody (Abcam) (1:500 dilution) overnight at 4°C, washed with HBSS and then incubated with Alexa Fluor 488 goat anti-rabbit antibody (Invitrogen, Inc.) (1:200 dilution) for 1 h at room temperature. Coverslips were washed with HBSS and mounted with Fluoroshield with DAPI (4′,6-diamidino-2-phenylindole) (Sigma-Aldrich). Microscopy was performed using a Leica DMI 4000B inverted confocal microscope using Leica Application Suite software. Imaging was done at 20× magnification using oil immersion. Each experimental condition was done in triplicate and >50 cells per replicate were recorded and fluorescence intensity was determined using ImageJ software (NIH, Bethesda, MD, United States).

### xCELLigence Real Time Cellular Analysis (RTCA)

RTCA DP (ACEA Biosciences) uses non-invasive electrical impedance monitoring to quantify cell proliferation, morphology change, and attachment quality in real-time. E-plates treated with fibronectin were used for all experiments. For each cell type, pilot studies determined adequate seeding density, expected CI, and growth rates. Briefly, after seeding of cells, plates were incubated in a 37°C incubator at 5% CO_2_. Cells were allowed to equilibrate in normal media until the cell index (CI) became stable and started to rise. Then, media were changed, drugs were added and changes in CI were monitored every 15 min. Data reported is from a minimum of two independent E-plate experiments (different passages of cells) and in each experiment, a given drug treatment was performed in triplicates and in wells at different locations to ensure that the data was not affected by positional effects.

### Seahorse Extracellular Flux Assay

Oxygen consumption rates (OCR) were measured using the Seahorse XFe24 Analyzer (Seahorse Biosciences, Agilent, United States). Seahorse Sensor cartridge was incubated overnight in XF calibrant at 37°C per manufacturer instructions. Briefly, on the day of the assay, hCAVSM cells were seeded on Seahorse 24-well plates at a density of 1.2 × 10^5^ cells per well. Seahorse media (Seahorse Biosciences, Agilent, United States) was used with Glucose (10 mM), Sodium pyruvate (1 mM), and Glutamine (2 mM) added fresh and warmed to 37°C. Cells were equilibrated to 37°C and the Seahorse XF Mitochondria Stress Test Kit was used per manufacturers protocol. Initially, appropriate pilot experiments were carried out to ensure correct concentrations of Oligomycin (1 μM) and FCCP (0.5 μM), and adequate cell seeding density (1.2 × 10^5^) for hCAVSMCs.

### Reactive Oxygen Species Detection (ROS)

Using the commercially available DCFDA/H2DCFDA Cellular Reactive Oxygen Species Detection Assay Kit (Abcam cat #ab113851), ROS was assayed in quadruplicates after background correction. NP-6A4 pretreated cells received daily medium change with NP-6A4 (1 μM) for 7 days prior to experiment. Briefly, 2.5 × 10^3^ hCAVSMC were seeded per well on a 96-well fibronectin treated plate, and allowed to adhere overnight. The next day, cells were washed and incubated in 40 μM DCFDA dye for 45 min at 37°C. Cells were then incubated in either media supplement with 1 μM doxorubicin or 1 μM NP-6A4 for 1 h. Cells were washed with media and read on fluorescent plate reader at 485/535 emission/excitation.

### Nitric Oxide Level Quantification Using DAF-FM Staining

We used the cell permeant reagent 4-Amino-5-Methylamino-2′,7′-Difluorofluorescein Diacetate (DAF-FM Diacetate, Thermo Fisher Scientific) to quantify NO generation in hCAECs. DAF-FM remains non-fluorescent until reacting with NO, at which time it forms a fluorescent benzotriazole. Briefly, endothelial cells were seeded on fibronectin treated slides and subsequently treated with either NP-6A4 (5 μM) or NP-6A4 (5 μM) + PD123319 (20 μM) for 24 h. Cells were then treated with 1 μM DAF-FM in DMEM (no phenol red) for 30 min at 37°C. Next, cells were washed twice with DMEM (no phenol red), and incubated at 37°C for 30 min to complete the DAF-FM reaction. Lastly, cells were fixed with ice-cold 4% paraformaldehyde and stained with Wheat Germ Agglutinin (WGA) conjugated with Alexa Fluor 555 (Thermo Fisher Scientific), in order to define cell borders. Slides were then visualized using a Leica DMI 4000B inverted confocal microscope using Leica Application Suite software. Imaging was done at 63× magnification using oil immersion. Each experimental condition was done in triplicate and >30 cells per replicate were recorded and fluorescence intensity was determined using ImageJ software (NIH, Bethesda, MD, United States).

### Western Blotting

Previously frozen cell pellets were homogenized with RIPA buffer [50 mM Tris–HCl (pH 8.0), 150 mM NaCl, 1% NP-40, 0.5% sodium deoxycholate, 1% SDS, 1× protease/phosphatase inhibitor cocktail (Thermo Scientific, Rockford, IL, United States)]. The homogenate was rotated for 30 min at 4°C and centrifuged at 12,000 *g* for 10 min, and the resultant supernatant was obtained for Western blotting. Protein concentrations were determined using the BCA assay (Pierce Biotechnology, Rockford, IL, United States), and 15 μg of protein in loading buffer was loaded onto SDS–PAGE gels. Next, proteins were transferred to nitrocellulose membranes, and all blots were incubated with Ponceau S (Sigma, St Louis, MO, United States) to validate equal loading across samples and lanes. Membranes were blocked overnight at 4°C in 5% fat free milk. Primary antibodies (Abcam) were rabbit polyclonal AT2R at 1:1000, mouse monoclonal eNOS at 1:500, rabbit polyclonal phospho-eNOS (Ser1177) at 1:1000. All antibodies were diluted in Tris-buffered saline + Tween-20 with 5% bovine serum albumin and applied to membrane overnight at 4°C. The following day, horseradish peroxidase-conjugated secondary antibody (1:1,000; Cell Signaling Technology, Danvers, MA, United States) was applied for 3 h at room temperature, and ECL substrate (Thermo Fisher) was applied immediately prior to exposure. Band densitometry was performed using Image Lab (Bio-Rad).

### Milliplex MAP Multi-Pathway Total and Phospho- Magnetic Bead 9-Plex Assay

hCAECs were grown to ∼70% confluence in T-75 flasks (Corning Inc, Corning, NY, United States), at which time cells were treated with either; NP-6A4 (5 μM),, or control medium for 24 h. PD123319 was added 20 min prior to NP-6A4 and a medium change with appropriate drugs was done after 12 h of treatment to ensure continuous drug exposure. Briefly, cells were placed on ice, washed twice with ice-cold PBS and lysed using 300 μl of manufacturers lysis buffer (with appropriate phosphatase inhibitors added fresh), scraped, collected, and agitated at 4°C for 15 min. Next, lysates were sterile filtered using EMD Millipore filter-tubes (Catalog # UFC30DV00 – Millipore Sigma, Burlington, MA, United States). Lysate was used for two separate assays, one for total proteins and a second for phosphorylated proteins (MILLIPLEX MAP Multi-Pathway Magnetic Bead Cell-Signaling 9-Plex; Millipore Sigma). The following probes were included as part of the kits: Total proteins and phosphorylated proteins for (phosphorylated residues in parenthesis); cAMP response element binding [CREB, (pS133)], c-Jun N-terminal kinase [JNK, (pT183/pY185)], nuclear factor kappa -light chain -enhancer of activated B cells [NFkB, (pS536)], p38 mitogen activated protein kinases [p38, (pT180/pY182)], p44/42 mitogen activated protein kinase [ERK1/2, (pT185/pY187)], protein kinase B [Akt, (pS473)], ribosomal protein S6 kinase beta [p70 S6K, (pT412)], signal transducer and activator of transcription 3 [STAT3, (pS727)], and signal transducer and activator of transcription 5 [STAT5A/B, (pY694/699)]. Assay was carried out following manufacturers protocol and fluorescence was assessed using xMAP^®^ technology on MAGPIX^®^ platform (Luminex, Austin, TX, United States). Mean Fluorescent Intensities were obtained in triplicate for each sample and compared between groups.

### Statistics

All analytical procedures were performed using Microsoft Excel v2011 (Microsoft, Redmond, WA, United States), Prism GraphPad 7 (GraphPad Software, CA, United States), and SAS 9.4 (SAS Institute Inc., Cary, NC, United States). Values are presented as means ± standard error of the mean (SEM). Significance for all analyses was set with a *p*-value of 0.05. Student’s *t*-test was used to assess between group differences for outcome measurements in studies involving two treatment groups. Differences in outcome variables for studies involving more than two treatment groups and/or timepoints were assessed by one-way or two-way ANOVA, as indicated in figure legends.

## Results

### NP-6A4 Treatment Increases Agtr2 mRNA and AT2R Protein in Human Cardiovascular Cells

**FIGURE 1:**
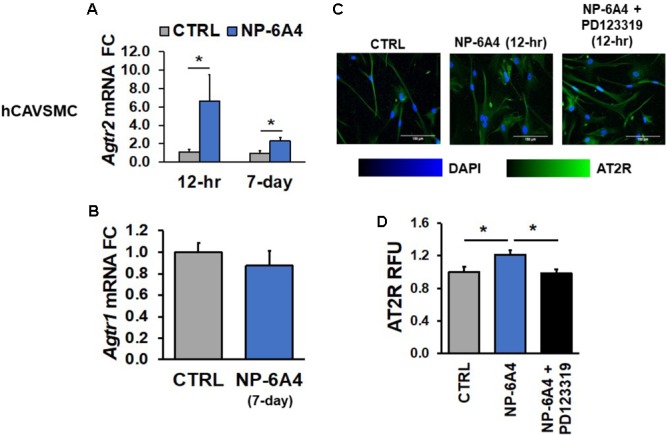
NP-6A4 increased *Agtr2* mRNA and AT2R protein expression in hCAVSMC. **(A)** 1 μM NP-6A4 for 12-h and 7-day increased *Agtr2* mRNA expression. **(B)** No change in *Agtr1* mRNA was detected after 1 μM NP-6A4 for 7-days. *n* = at least three biological replicates and PCR replicates, respectively, for these experiment. **(C)** Representative images of hCAVSMCs treated with 1 μM NP-6A4 for 12-h. Blue indicates DAPI stained nuclei and green AT2R. Merged images are shown for all experimental conditions. **(D)** Quantification of relative fluorescent units (RFU) shows increased expression of AT2R protein after 1 μM NP-6A4 treatment for 12-h and 20 μM PD123319 prevented this increase. Immunofluorescent experiments consisted of quantification of >50 cells from triplicates per experiment. ^∗^Indicates *p* < 0.05 using One-way ANOVA. hCAVSMC, human coronary artery vascular smooth muscle cell; FC, fold change.

To further elucidate the potential protective mechanisms of NP-6A4 in human cardiovascular cells, we assessed the effects of NP-6A4 treatment on hCAVSMC, hCAEC, and hUVECs. *Agtr2* mRNA has a reported half-life of ∼18 h (Kijima et al., 1995), implying that any change should be detectable within the 12-h time window. In hCAVSMCs, NP-6A4 (1 μM) increased *Agtr2* mRNA (sixfold, *p* < 0.05) after a 12-h treatment (**Figure 1A**) and the effect remained after 7-days of 1 μM NP-6A4 (**Figure 1A**), while no change in AT1R expression was overserved (**Figure 1B**). Given that the half-life of *Agtr2* mRNA is in the 12–18-h range, we assessed AT2R protein expression after 12-h of NP-6A4 treatment. AT2R protein increase was detected using immunofluorescent staining after 12-h of NP-6A4 treatment, while pretreatment with AT2R-antagonist PD123319 attenuated NP-6A4-mediated increase in AT2R protein levels in hCAVSMCs (**Figures 1C,D**). These observations indicated that NP-6A4 acting through the AT2R induces a positive feedback loop to increase *Agtr2* mRNA and AT2R protein expression and signaling.

**FIGURE 2:**
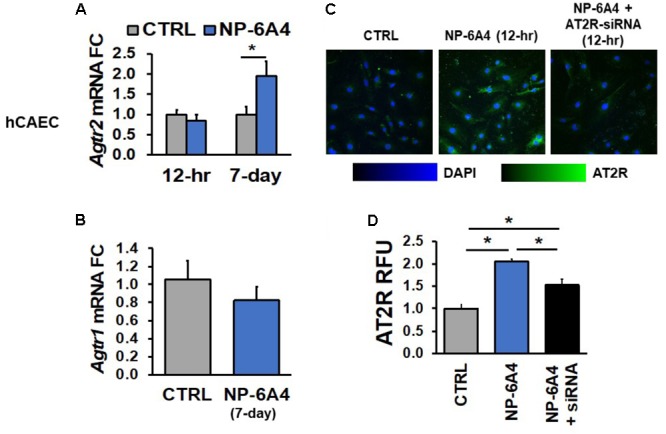
NP-6A4 increased *Agtr2* mRNA and AT2R protein expression in hCAECs. **(A)** 1 μM NP-6A4 for 12-h showed no difference in *Agtr2* mRNA, while 7-day treatment increased *Agtr2* mRNA expression twofold. **(B)** No change in *Atgr1* was detected after 1 μM NP-6A4 for 7-days. *n* = at least three biological replicates and PCR replicates, respectively, for these experiment. **(C)** Representative images of hCAECs treated with 1 μM NP-6A4 for 12-h. Blue indicates DAPI stained nuclei and green AT2R. Merged images are shown for all experimental conditions. **(D)** Quantification of relative fluorescent units (RFU) shows increased expression of AT2R protein after 1 μM NP-6A4 treatment for 12-h. AT2R-siRNA prevented NP-6A4-induced increase in AT2R expression. Immunofluorescent experiments consisted of quantification of >50 cells from triplicates per experiment. ^∗^Indicates *p* < 0.05 using One-way ANOVA. hCAEC, human coronary artery endothelial cell; FC, fold change.

Next, we tested whether NP-6A4-mediated increases in *Agtr2* mRNA and AT2R protein also occurs in human endothelial cells using hCAECs and hUVECs. While a 12-h NP-6A4 (1 μM) treatment of hCAECs (**Figure 2A**) was not sufficient to increase *Agtr2* mRNA, a chronic treatment for 7 days with NP-6A4 (1 μM) resulted in a twofold increase in *Agtr2* mRNA in these cells (**Figure 2A**). However, a 12-h treatment with NP-6A4 (1 μM) was sufficient to increase AT2R protein levels by twofold in hCAECs (**Figures 2C,D**). Thus, the time course to increase *Agtr2* mRNA by NP-6A4 differed between hCAVSMCs and hCAECs. To confirm further that the effects of NP-6A4 are mediated through the AT2R protein expression, we transfected hCAECs with an anti-AT2R siRNA prior to the treatment with NP-6A4 (1 μM). NP-6A4 treatment did not increase AT2R protein levels in hCAECs transfected with anti-AT2R siRNA (**Figures 2C,D**) indicating that NP-6A4 needs AT2R protein expression to activate a positive feedback loop.

**FIGURE 3:**
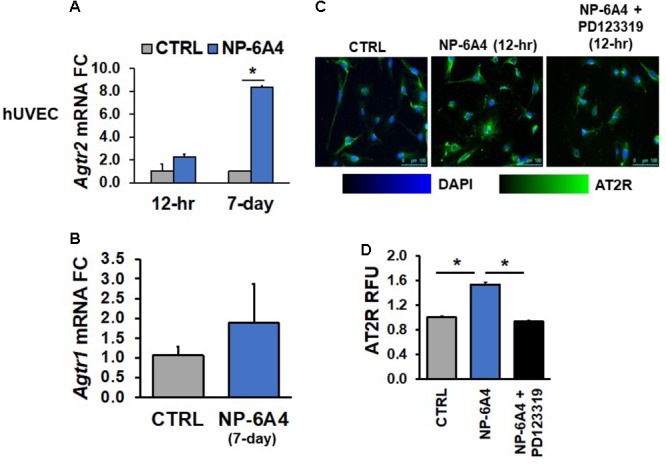
NP-6A4 increased *Agtr2* mRNA and AT2R protein expression in hUVEC. **(A)** 1 μM NP-6A4 for 12-h showed no difference in *Agtr2* mRNA, while 7-day treatment increased *Agtr2* mRNA expression eightfold. **(B)** No change in *Atgr1* was detected after 1 μM NP-6A4 for 7-days. *n* = at least three biological replicates and PCR replicates, respectively, for these experiment. **(C)** Representative images of hUVECs treated with 1 μM NP-6A4 for 12-h. Blue indicates DAPI stained nuclei and green AT2R. Merged images are shown for all experimental conditions. **(D)** Quantification of relative fluorescent units (RFU) shows increased expression of AT2R protein after 1 μM NP-6A4 treatment for 12-h and 20 μM PD123319 prevented this increase. Immunofluorescent experiments consisted of quantification of >50 cells from triplicates per experiment. ^∗^Indicates *p* < 0.05 using One-way ANOVA. hUVEC, human umbilical vein endothelial cell; FC, fold change.

Similar findings were also observed in hUVECs; *Agtr2* mRNA levels did not change in hUVECs after 12-h NP-6A4 (1 μM) treatment, whereas 7-days of NP-6A4 treatment resulted in an almost eightfold increase in *Agtr2* mRNA levels in these cells (**Figure 3A**). However, as in hCAECs, a 12-h NP-6A4 (1 μM) treatment was sufficient to increase AT2R protein in hUVECs (**Figures 3C,D**). Moreover, pre-treatment with AT2R antagonist PD123319 before the addition of NP-6A4, attenuated NP-6A4-induced increase in AT2R protein expression in hUVECs. Collectively, these observations suggest that in human coronary artery VSMCs and ECs, NP-6A4 increases *Agtr2* mRNA and AT2R protein expression, albeit the time course for this effect can be cell-specific.

Previous studies have indicated that AT2R agonist CGP42112A suppresses AT1R mRNA expression (Yang et al., 2012). Therefore, we tested whether NP-6A4 treatment modulated expression of the AT1R mRNA in human coronary artery VSMCs and ECs. We did not observe any changes in *Agtr1* mRNA after 7-days of NP-6A4 (1 μM) treatment in hCAVSMCs, hCAECs and hUVECs (**Figures 1B, 2B, 3B**).

### NP-6A4 Is Not Cytotoxic, Promotes Favorable Growth Conditions, and Enhances Mitochondrial Energetics in hCAVSM Cells

**FIGURE 4:**
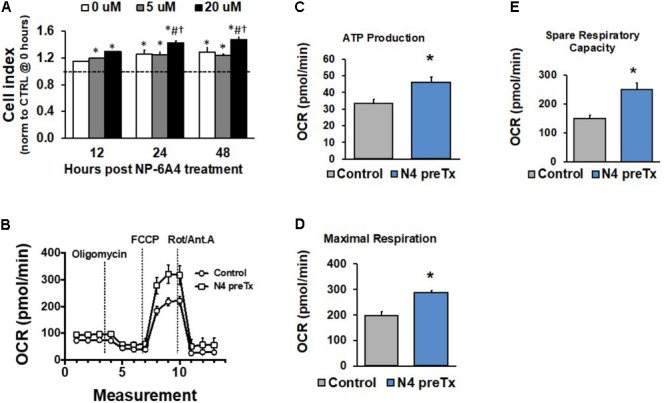
NP-6A4 increased hCAVSMC growth properties and mitochondrial energetics. **(A)** Cell Index in cells pretreated with NP-6A4 for 7-days and then treated with increasing doses of acute NP-6A4 from 0 to 20 μM. All doses increased CI compared to untreated (CTRL) cells. Data were analyzed using a two-way ANOVA with dose and time serving as independent variables. ^∗^indicates *p* < 0.05 vs. CTRL cells at baseline. ^#^Indicates *p* < 0.05 vs. 0 μM at same time point. ^†^Indicates *p* < 0.05 vs. 5 μM at the same time point. *n* = 4 per group. **(B)** OCR increased after 7-days of 1 μM NP-6A4 treatment measured using the Seahorse Extracellular Flux Assay. **(C)** ATP production was increased after NP-6A4 treatment. **(D)** Maximal respiration was increased after NP-6A4 treatment. **(E)** Spare respiratory capacity was increased after NP-6A4 treatment. *n* = 3 per group. ^∗^Indicates *p* < 0.05 using One-way ANOVA. hCAVSMC, human coronary artery vascular smooth muscle cell.

We reported previously that when hCAVSMCs were grown in medium 231 in nutrient deficient conditions generated by the absence of Smooth Muscle Growth Supplement (SMGS), NP-6A4 (300 nM) could improve their viability significantly better than β1 adrenergic receptor blockers (atenolol, metoprolol, carvedilol, and nebivolol), losartan and AT2 receptor peptide agonist CGP42112A (Mahmood and Pulakat, 2015). To determine if treatment with higher concentrations of NP-6A4 would exert any cytotoxic effects on hCAVSMCs, we investigated how increasing concentrations of NP-6A4 (1–20 μM) would modulate cell index (CI) of hCAVSMCs grown in nutrient-rich medium (medium 231 + SMGS) for a period of 48 h using the Real Time Cell Analyzer (RTCA). CI is a quantitative measure of cell number, cell size and cell adhesion. Since we observed that a 7-day treatment of hCAVSMCs grown in nutrient rich medium with NP-6A4 (1 μM) did not exhibit any negative growth effects and they retained an increase in *Agtr2* mRNA (**Figure 1A**), we pre-treated hCAVSMCs for 7 days with NP-6A4 (1 μM) before seeding on the E-plate for RTCA assay. hCAVSMCs that were not pre-treated with NP-6A4 were used as controls in the experiment and the data in **Figure 4A** is presented after normalizing to these control (CTRL) cells (dotted line on **Figure 4A**). At all points during the time course, all concentrations of NP-6A4 induced a steady increase in CI compared to CTRL cells (**Figure 4A**). The best effect was observed with 20 μM NP-6A4 that increased the CI as early as 12-h after exposure (**Figure 4A**, *p* < 0.05). Additionally, NP-6A4 pre-treatment alone was sufficient to significantly increase CI vs. CTRL cells after 24 and 48-h treatments (**Figure 4A**, *p* < 0.05). Only at 12 h time point, NP-6A4 pre-treated cells did not exhibit significant change in CI in the absence of NP-6A4. Thus, hCAVSMCs did not exhibit any cytotoxic effects when they were exposed to NP-6A4 for up to nine days (7 days pre-treatment with 1 μM NP-6A4 and 2 days additional exposure to 0, 5, and 20 μM doses of NP-6A4), and exhibited favorable growth response to NP-6A4 as evidenced by their increased CI (**Figure 4A**, *p* < 0.05). Moreover, the increase in CI by 20 μM dose of NP-6A4 was significantly higher than the increase in CI seen with the 5 μM dose of NP-6A4 at both the 24 and 48 h timepoint, indicating that NP-6A4 increased the CI of hCAECs in a dose-dependent manner.

Given the discovery and characterization of a functional mitochondrial renin-angiotensin system (RAS) (Abadir et al., 2011) and relatively little is known regarding AT2R receptor activity at the level of mitochondria, we sought to investigate the ability of NP-6A4 to modulate mitochondrial energetics, specifically oxygen consumption rates, using the Seahorse Extracellular Flux Assay. The XF Cell Mito Stress Test allows for measurement of key parameters of mitochondrial function through direct detection of oxygen consumption rates of cultured cells. hCAVSM cells treated with 1 μM NP-6A4 for 1-week showed an increase oxygen consumption rate (**Figure 4B**), ATP production (**Figure 4C**, *p* < 0.05), maximal respiration (**Figure 4D**, *p* < 0.05), and spare respiratory capacity (**Figure 4E**, *p* < 0.05).

**FIGURE 5:**
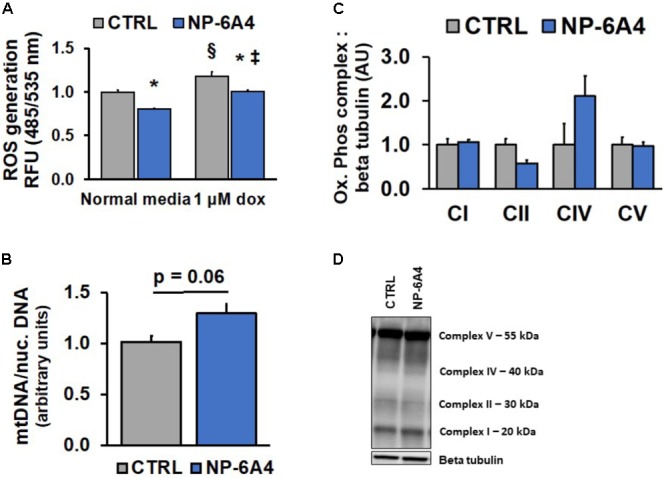
NP-6A4 reduces reactive oxygen species while not altering mitochondrial number in hCAVSMCs. **(A)** Cellular ROS was increased with 1 μM Doxorubicin, while 1 μM NP-6A4 reduced ROS generation in both normal media and 1 μM Doxorubicin. *n* = 4. **(B)** Mitochondrial DNA was non-significantly increased after 7 days of NP-6A4 treatment. *n* = 4. **(C)** Oxidative phosphorylation complexes quantification of Western Blot images. *n* = 3. **(D)** Representative image of the Western blot for oxidative phosphorylation complexes after 7-days of NP-6A4 treatment. ^∗^Indicates *p* < 0.05 vs. CTRL using two-tailed Student’s *t*-test. ^§^Indicates *p* < 0.05 vs. CTRL cells (normal media). ^‡^Indicates *p* < 0.05 vs. NP-6A4 preTx cells (normal media). OCR, oxygen consumption rate; hCAVSMC, human coronary artery vascular smooth muscle cell.

The role of Ang II in activating NADPH oxidase and increasing reactive oxygen species is well established. It is unclear how activation of AT2R modulates reactive oxygen species in hCAVSMCs. Given that mitochondria are important sources of reactive oxygen species (ROS) (Jensen, 1966; Cortassa et al., 2017), we investigated how treatment with NP-6A4 (1 μM) for 7-days modulates ROS levels in hCAVSMCs. The cell-permeant 2′,7′-dichlorodihydrofluorescein diacetate (H2DCFDA) is a chemically reduced form of fluorescein that can be used as an indicator for ROS in cells. Therefore, we stained NP-6A4-pretreated and untreated hCAVSMC cells with DCFDA to detect changes in ROS levels. The ROS levels were significantly reduced in NP-6A4 pre-treated hCAVSMCs compared to untreated (CTRL) hCAVSMCs (**Figure 5A**, *p* < 0.05).

Doxorubicin (DOXO), a chemotherapeutic agent widely used in cancer treatment, causes significant increases in ROS (Kim et al., 2006; Asensio-Lopez et al., 2017). Since DOXO treatment is known to induce vascular toxicity (Bielak-Zmijewska et al., 2014), we investigated whether DOXO treatment would increase ROS in hCAVSMC. Treatment with 1 μM DOXO caused an increase in ROS in CTRL hCAVSMCs compared to untreated cells, however, NP-6A4 pre-treatment suppressed this effect (**Figure 5A**, *p* < 0.05). Collectively, these observations suggest that NP-6A4 treatment is effective in reducing ROS levels in hCAVSMCs. We did not see a significant change in mtDNA content in response to NP-6A4 treatment, although a trend was observed (**Figure 5B**, *p* = 0.06). Moreover, Immunoblotting analysis of all the oxidative phosphorylation protein complexes in whole cell lysates of NP-6A4-treated and CTRL hCAVSMCs revealed no significant differences in complex I (CI), complex II (CII), complex V (CV), or complex IV (CIV) were observed (**Figures 5C,D**). Taken together, these data suggest that the increased OCR concomitant with decreased ROS observed after chronic NP-6A4 treatment is likely not the result of increased quantity, but likely a change in the quality and efficacy of the existing mitochondria.

### Endothelial Cell Response to NP-6A4 Treatment

**FIGURE 6:**
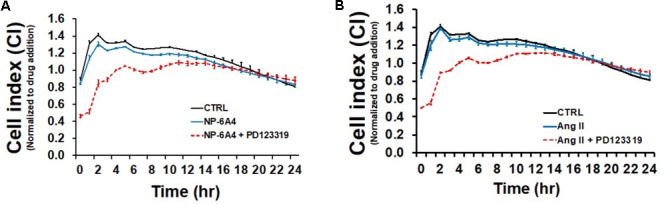
Growth promoting effects of NP-6A4 are similar to Angiotensin II in hCAECs. **(A)** NP-6A4 did not increase CI in hCAEC, while AT2R-agonist PD123319 decreased CI initially but stabilized within 24-h. **(B)** Likewise, Ang II did not change CI compared to normal medium, while PD123319 attenuated CI at 6 h post treatment but stabilized within 24-h. *n* = 4. Control data is the same in A and B. CI, cell index; Ang II, angiotensin II; hCAECs, human coronary artery endothelial cells.

**FIGURE 7:**
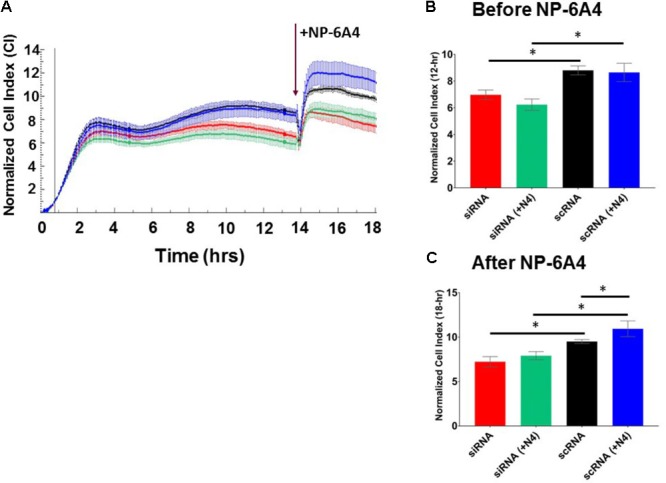
AT2R siRNA inhibited growth properties (CI) of hCAEC both in the absence and presence of NP-6A4. **(A)** Cell Index curve depicting change in cell index (CI). Arrow indicates addition of NP-6A4. **(B)** Differences in CI after 12 h of treatment with anti-AT2R siRNA (labeled siRNA) or siRNA-Scrambled (scRNA). At this point none of the cells had been treated with NP-6A4. *n* = 4 per group. **(C)** Differences in CI approximately 4-h after the addition of NP-6A4 (20 μM). *n* = 4 per group. ^∗^Indicates *p* < 0.05 vs. CTRL using one-way ANOVA at the indicated time points.

Endothelial cells have a critical role in maintaining vascular health because they play a crucial role in maintaining bioavailability of nitric oxide and proper vasodilation (Prieto et al., 2014; Khaddaj Mallat et al., 2017). Moreover, EC cell death underlies atherosclerotic plaque formation and destabilization (Di et al., 2017). First, we explored the growth characteristics of hCAECS using the RTCA system in an attempt to understand NP-6A4 and its ability to alter general cellular growth characteristics. Interestingly, NP-6A4 and Ang II treatment did not augment the cell index in hCAECs (**Figures 6A,B**). However, the cell index of hCAECs (**Figures 6A,B**) was significantly decreased (*p* < 0.05) when treated with the AT2R antagonist PD123319 (20 μM) before treating with NP-6A4 (1 μM) and Ang II (500 nM). This suggests that, to some degree, that AT2R receptor activation plays a role in human coronary artery endothelial cells response in terms of cell proliferation, morphology change, and attachment quality. PD123319 is reported to have off-target effects and its role in increasing abdominal aortic aneurisms in response to Ang II is not via AT2R (Daugherty et al., 2013). Therefore, it is conceivable that use of 20 μM PD123319 in our experiments could have exerted some off-target effects that contributed to the eventual increase in the CI of hCAEC in the presence of PD123319. To further confirm that NP-6A4 acted through the AT2R in hCAECs, we transfected the cells with anti-AT2R siRNA for 14 h before adding NP-6A4 (**Figure 7**). Transfection with Scrambled siRNA was used as control. Interestingly, the CI of hCAECs transfected with anti-AT2R siRNA (labeled siRNA) was found to be lower than those transfected with siRNA-Scrambled (labeled scRNA) before the addition of NP-6A4 (**Figures 7A,B**). Thus, suppression of AT2R expression was detrimental to the CI of hCAECs. Addition of NP-6A4 significantly increased the CI of hCAECs transfected with scRNA compared to those that did not receive NP-6A4 treatment (**Figures 7A,C**). However, addition of NP-6A4 did not significantly change the CI of hCAECs transfected with anti-AT2R siRNA (labeled siRNA + N4) compared to those that did not receive NP-6A4 treatment (labeled siRNA) (**Figures 7A,C**). Thus, the effect of NP-6A4 on the CI of hCAECs was abolished by anti-AT2R siRNA.

**FIGURE 8:**
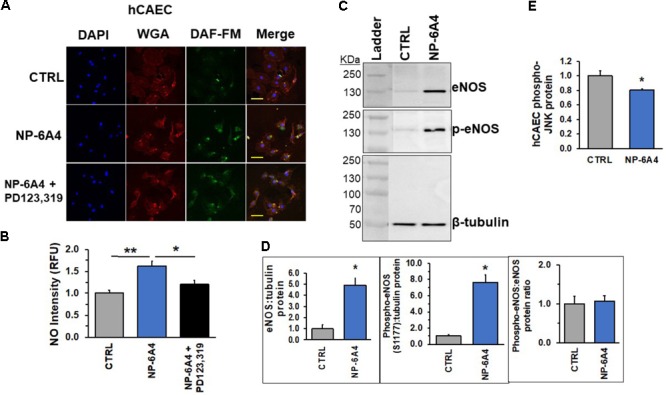
NP-6A4 promotes cellular protective signaling in hCAECs. **(A)** Representative images of DAF-FM diacetate staining in hCAEC. Blue DAPI images show nuclei, Red wheat germ agglutinin (WGA) images show endothelial cell membrane, Green DAF-FM images show presence of detectable nitric oxide, and lastly, merged images show al three colors on one image. *n* = 3. **(B)** Quantification of detectable NO showed that 5 μM NP-6A4 for 24 h increased NO, while PD123319 pretreatment prevented the increase. Fluorescent staining experiments consisted of quantification of >50 cells from triplicates. **(C)** Representative western blot images of eNOS and phospho-eNOS protein. **(D)** Quantification of eNOS and phosphos-eNOS show increased expression of eNOS after 12-h of 1 μM NP-6A4 treatment. The total to phospho-eNOS ratio was not affected. *n* = 4. **(E)** 5 μM NP-6A4 of hCAEC for 24-h decreased phosphorylated-JNK as determined by the Milliplex MAP Multi-Pathway Total and Phospho- Magnetic Bead 9-Plex Assay. ^∗∗^Indicates difference from CTRL and ^∗^Indicates difference from NP-6A4-treated at *p* < 0.05 using One-way ANOVA. Yellow scale bar = 100 microns. hCAEC, human coronary artery endothelial cell; DAF-FM = 4-Amino-5-Methylamino-2′,7′-Difluorofluorescein Diacetate.

AT2R activation is reported to increase eNOS expression and NO bioavailability in human endothelial cells (Peluso et al., 2018). To determine whether NP-6A4 exerted similar effects in hCAECs, we investigated expression and phosphorylation levels of eNOS and NO generation in hCAECs treated with 5 μM NP-6A4. Preliminary time course experiments confirmed that 24-h NP-6A4 (5 μM) treatment elicited the largest increase (and detectable) in NO compared to untreated cells (**Supplementary Figure 1**). Therefore, hCAE cells treated with 5 μM NP-6A4 for 24 h were stained with the cell permeant stain, DAF-FM. NO was significantly increased (*p* < 0.05) compared to control (**Figures 8A,B**). Interestingly, pretreatment with PD123319 (20 μM) prior to NP-6A4 inhibited the increase in NO intensity. This data suggests that AT2R activation is upstream of NO release and imperative for the increased NO after agonist treatment. To confirm the increase in NO levels, we hypothesized that eNOS protein levels would be increased. Immunoblotting of the lysates from hCAECs with or without NP-6A4 (1 μM) treatment for 12 h showed that both total and phosphorylated eNOS proteins were increased in NP-6A4-treated hCAECs (**Figures 8C,D**, *p* < 0.05).

Lastly, in an attempt to better understand NP-6A4-AT2R-induced downstream signaling pathways, we analyzed changes in intra-cellular kinases implicated in metabolism, cell growth, physiology and pathology of hCAECs. Utilizing the Milliplex MAP multiple-pathway total and phosphorylated cell signaling 9-plex kits, we analyzed the following targets: CREB, JNK, NFkB, p38, ERK1/2, Akt, p70 S6 kinase, STAT3, and STAT5. To our surprise, NP-6A4 treatment did not alter the total protein for any of the targets in this passage of hCAECs (**Figure 8E** and **Supplementary Figure 2**). However, the phospho-protein assay showed that phospho-JNK showed a significant decrease compared to CTRL cells (*p* < 0.05, **Figure 8E**). Taken together, our data in hCAECs confirms that AT2R activation via NP-6A4 is sufficient to promote cell growth, viability, and increase NO release. Although the exact mechanisms remain unresolved, future experiments will aim to uncover the intricacies involved in this response.

## Discussion

Here we show for the first time that the AT2R peptide agonist, NP-6A4, increases the expression levels of both *Agtr2* mRNA and AT2R protein and activates a positive feed-back loop for AT2R signaling in hCAVSMCs and endothelial cells *in vitro*. As a direct result, downstream signaling is augmented, promoting an enhancement of NO signaling and mitochondrial bioenergetics. Important to the cardiovascular system, AT2R receptors are expressed on both endothelial and vascular smooth muscle cells and have been identified in rat small resistance arteries (Nora et al., 1998; Matrougui et al., 1999), and within mouse coronary arteries (Akishita et al., 2000; Wu et al., 2002). In human pulmonary microvascular endothelial cells (HPMEC), *Agtr2* mRNA was not detected under normal conditions or in response to LPS injury (Li et al., 2015), while *Agtr1* mRNA was increased after LPS. While AT2R-agonists, specifically Compound 21, and to a lesser extent CGP42112, have been reported to confer beneficial effects in *ex vivo* and *in vivo* models (Ewert et al., 2003; Bosnyak et al., 2010; Rehman et al., 2012; Sampson et al., 2016), there have been no reports of either AT2R agonist having the ability to augment AT2R expression levels either at the mRNA or protein levels.

Using NP-6A4, we consistently demonstrate an increase in *Agtr2* mRNA, ranging from two- to eightfold in cardiovascular cells. Interestingly, hCAVSMCs showed the increase after short (12-h) and long-term (7-days) of NP-6A4 treatment. This is in contrast to both endothelial cell types studied, whereby 12-h NP-6A4 treatment was insufficient to augment mRNA levels, but in both hCAEC and hUVECs, the 7-day treatment showed a significant increase. Collectively, this suggests that the activation of AT2R and subsequent alterations in transcription and translation are uniquely regulated in hECs compared to hVSMCs. Although 12-h of NP-6A4 increased AT2R protein in all three cell types, hCAECs experienced the largest fold-increase compared to control, with a ∼2.0-fold increase. Albeit no AT2R agonist has been shown to increase AT2R, in a model of ischemia-reperfusion in isolated rat hearts, the AT1R-blocker Candesartan offered cardio protection through increased AT2R receptors, providing alternative means whereby an increase in AT2R in achieved and confers cardioprotection (Moudgil et al., 2002).

To our knowledge, our data is the first to show that AT2R upregulation via an AT2R agonist increased cellular oxygen consumption rates in hCAVSMCs. Historically, RAS has been defined as a circulating system involved in the maintenance of blood pressure through renal and vascular mechanisms (Gwathmey et al., 2012). Notwithstanding, recent studies have shown that local and intracellular RAS within specific cellular compartments may be responsible for numerous regulatory effects of Ang II (De Mello, 1994; Filipeanu et al., 2001; Re, 2007). Nuclear and mitochondrial RAS are involved with NO production (Gwathmey et al., 2012), and mitochondrial respiration (Abadir et al., 2011). Our data is in contrast to the current literature that shows in isolated mitochondria, decreased oxygen consumption after treatment with the AT2R-agonist CGP421140 (Abadir et al., 2011). Additionally, in HL-1 – mouse cardiomyocytes, Ang II decreased oxygen consumption rates, supporting the notion that activation of RAS, either at the mitochondria or the whole cell level, may decrease oxygen consumption (Kim et al., 2017). There are differences between these reports and ours worth noting. Firstly, the use of isolated mitochondria vs. whole cell respiration measurements, and second, the AT2R agonist used, CGP421140 and Ang II vs. NP-6A4. Additionally, the longer duration of treatment in our studies likely had an effect on the ability of NP-6A4 to augment mitochondrial respiration. Furthermore, we cannot rule out that the source of cellular respiration is solely via the mitochondria, as others suggest that oxygen consumption originating at the cell surface involving cellular NADH lends support to non-mitochondrial sources of oxygen consumption (Herst and Berridge, 2007). Lastly, our data suggests that the increase in oxygen consumption is not due to an increase in mitochondrial quantity, given that mtDNA and mitochondrial oxidative phosphorylation complexes were not significantly increased.

Reactive oxygen species (ROSs) are a class of gaseous signaling molecules related to NO. It is well accepted that the uncoupling of the mitochondrial respiratory chain and activation by cytosolic NADPH-oxidase contribute to ROS generation (Bellin et al., 2006). Doxorubicin (DOXO) is an anthracycline chemotherapeutic drug that is known to increase ROSs (Minotti et al., 2004; Kim et al., 2006) in various cell types including cardiomyocytes (Kim et al., 2006) and endothelial cells (Sonowal et al., 2018). Furthermore, the widely used chemotherapeutic anthracycline, DOXO, is known to attenuate beneficial eNOS signaling and resultant increase in NO release from endothelial cells (Sonowal et al., 2018). Our observation that DOXO treatment increases ROS in hCAVSMCs is in agreement with previous studies on the effect of DOXO treatment on other cell types. However, our observation that NP-6A4 treatment could reduce ROS levels in hCAVSMCs treated with DOXO suggests that NP-6A4 may exert protective effects on CAVSMCs in the presence of DOXO treatment.

It has been reported that Ang II, acting through AT2R, induces apoptosis in hUVECs and implicated AT2R-signaling in their dysfunction (Dimmeler et al., 1997). Alternatively, others have shown that in mouse VSMCs, AT2R activation neither increased eNOS phosphorylation via ser1177, nor changed plasma NO in ApoE^(-/-)^ mice (Li et al., 2016). Further evidence shows that some of the beneficial effects of AT2R are mediated via bradykinin and nitric oxide (NO) system (Liu et al., 1997; Gohlke et al., 1998). NP-6A4 treatment did not enhance the growth characteristics of hCAECs. This is in contrast to the hCAVSMC response to NP-6A4 (**Figure 4A**), that showed an increased cell index and thus a positive response to cell growth conditions, with doses up to 20 μM. However, treatment with the AT2R-agonist PD123319 attenuated the CI of hCAECs, indicating that inhibition of AT2R is detrimental to the cells. Taken together, this suggests that hCAVSMCs and hCAECs demonstrate differential response to activation of AT2R signaling by NP-6A4, with the latter showing no response in cell index.

Endothelial cells lining the coronary artery are essential for regulation of coronary blood flow and overall cardiac functions, implicating them in injury and disease conditions. Given that hCAECs are known to have higher expression of AT1R compared to AT2R (Li et al., 1999), the ability of NP-6A4 to increase the expression of AT2R mRNA and protein is a critical feature of this drug’s pharmacodynamics. Our data is the first to show that using an AT2R agonist, eNOS and NO levels can be augmented in hCAECs. In one study, NO release was improved via activation of the bradykinin system via AT2R stimulation (Harada et al., 2002). However, these experiments were performed in isolated aortic endothelial cells from spontaneously hypertensive WKY rats and through the use of Losartan to block AT1R (which allow for increased AT2R activation). Notwithstanding, this data lends support to our findings using coronary artery endothelial cells, that increased AT2R-signaling can augment NO-signaling.

These studies provide the first evidence that an AT2R agonist can increase the expression of *Agtr2* mRNA and AT2R protein in cardiovascular cells. Importantly, downstream cellular protective AT2R signaling is augmented while NP-6A4 does not cause cell toxicity. Vascular smooth muscle cells responded via increased cellular respiration, suggesting that NP-6A4-AT2R signaling can enhance mitochondrial energy dynamics. Likewise, NP-6A4 doses up to 20 μM did not cause any growth arresting side effects. Endothelial cells responded to NP-6A4-induced AT2R activation via increased eNOS signaling and higher levels of NO.

## Author Contributions

RT and LP developed conceptual ideas and design. RT carried out the experiments, data analysis, figure generation, and wrote the manuscript. AB carried out Milliplex MAP Multi-Pathway Total and Phospho-Magnetic Bead 9-Plex Assay, data analysis, figure generation, and manuscript editing. All authors approved the final version of the manuscript.

## Conflict of Interest Statement

The authors declare that the research was conducted in the absence of any commercial or financial relationships that could be construed as a potential conflict of interest. The reviewer PB and handling Editor declared their shared affiliation.

## References

[B1] Abadir P. M. Foster D. B. Crow M. Cooke C. A. Rucker J. J. Jain A. (2011). Identification and characterization of a functional mitochondrial angiotensin system. *Proc. Natl. Acad. Sci. U.S.A.* 108 14849–14854. 10.1073/pnas.1101507108 21852574 PMC3169127

[B2] Akishita M. Horiuchi M. Yamada H. Zhang L. Shirakami G. Tamura K. (2000). Inflammation influences vascular remodeling through AT2 receptor expression and signaling. *Physiol. Genomics* 2 13–20. 10.1152/physiolgenomics.2000.2.1.13 11015577

[B3] Ali Q. Wu Y. Hussain T. (2013). Chronic AT2 receptor activation increases renal ACE2 activity, attenuates AT1 receptor function and blood pressure in obese Zucker rats. *Kidney Int.* 84 931–939. 10.1038/ki.2013.193 23823602 PMC4091804

[B4] Altarche-Xifro W. Curato C. Kaschina E. Grzesiak A. Slavic S. Dong J. (2009). Cardiac c-kit+AT2+ cell population is increased in response to ischemic injury and supports cardiomyocyte performance. *Stem Cells* 27 2488–2497. 10.1002/stem.171 19591228

[B5] Asensio-Lopez M. C. Soler F. Pascual-Figal D. Fernandez-Belda F. Lax A. (2017). Doxorubicin-induced oxidative stress: the protective effect of nicorandil on HL-1 cardiomyocytes. *PLoS One* 12:e0172803. 10.1371/journal.pone.0172803 28245258 PMC5330507

[B6] Axelsson A. Iversen K. Vejlstrup N. Ho C. Norsk J. Langhoff L. (2015). Efficacy and safety of the angiotensin II receptor blocker losartan for hypertrophic cardiomyopathy: the INHERIT randomised, double-blind, placebo-controlled trial. *Lancet Diabetes Endocrinol.* 3 123–131. 10.1016/S2213-8587(14)70241-4 25533774

[B7] Axelsson A. Iversen K. Vejlstrup N. Ho C. Y. Havndrup O. Kofoed K. F. (2016). Functional effects of losartan in hypertrophic cardiomyopathy-a randomised clinical trial. *Heart* 102 285–291. 10.1136/heartjnl-2015-308343 26661322

[B8] Bellin C. de Wiza D. H. Wiernsperger N. F. Rosen P. (2006). Generation of reactive oxygen species by endothelial and smooth muscle cells: influence of hyperglycemia and metformin. *Horm. Metab. Res.* 38 732–739. 10.1055/s-2006-955084 17111300

[B9] Bielak-Zmijewska A. Wnuk M. Przybylska D. Grabowska W. Lewinska A. Alster O. (2014). A comparison of replicative senescence and doxorubicin-induced premature senescence of vascular smooth muscle cells isolated from human aorta. *Biogerontology* 15 47–64. 10.1007/s10522-013-9477-9 24243065 PMC3905196

[B10] Borghi C. Force S. T. Rossi F. Force S. I. F. T. (2015). Role of the renin-angiotensin-aldosterone system and its pharmacological inhibitors in cardiovascular diseases: complex and critical issues. *High Blood Press. Cardiovasc. Prev.* 22 429–444. 10.1007/s40292-015-0120-5 26403596

[B11] Bosnyak S. Welungoda I. K. Hallberg A. Alterman M. Widdop R. E. Jones E. S. (2010). Stimulation of angiotensin AT2 receptors by the non-peptide agonist, Compound 21, evokes vasodepressor effects in conscious spontaneously hypertensive rats. *Br. J. Pharmacol.* 159 709–716. 10.1111/j.1476-5381.2009.00575.x 20128808 PMC2828034

[B12] Chow B. S. Koulis C. Krishnaswamy P. Steckelings U. M. Unger T. Cooper M. E. (2016). The angiotensin II type 2 receptor agonist Compound 21 is protective in experimental diabetes-associated atherosclerosis. *Diabetologia* 59 1778–1790. 10.1007/s00125-016-3977-5 27168137

[B13] Cortassa S. Sollott S. J. Aon M. A. (2017). Mitochondrial respiration and ROS emission during beta-oxidation in the heart: an experimental-computational study. *PLoS Comput. Biol.* 13:e1005588. 10.1371/journal.pcbi.1005588 28598967 PMC5482492

[B14] Curato C. Slavic S. Dong J. Skorska A. Altarche-Xifro W. Miteva K. (2010). Identification of noncytotoxic and IL-10-producing CD8+AT2R+ T cell population in response to ischemic heart injury. *J. Immunol.* 185 6286–6293. 10.4049/jimmunol.0903681 20935205

[B15] Daugherty A. Rateri D. L. Howatt D. A. Charnigo R. Cassis L. A. (2013). PD123319 augments angiotensin II-induced abdominal aortic aneurysms through an AT2 receptor-independent mechanism. *PLoS One* 8:e61849. 10.1371/journal.pone.0061849 23593499 PMC3625148

[B16] De Mello W. C. (1994). Is an intracellular renin-angiotensin system involved in control of cell communication in heart? *J. Cardiovasc. Pharmacol.* 23 640–646. 10.1097/00005344-199404000-00018 7516016

[B17] Di M. Wang L. Li M. Zhang Y. Liu X. Zeng R. (2017). Dickkopf1 destabilizes atherosclerotic plaques and promotes plaque formation by inducing apoptosis of endothelial cells through activation of ER stress. *Cell Death Dis.* 8:e2917. 10.1038/cddis.2017.277 28703797 PMC5550842

[B18] Dimmeler S. Rippmann V. Weiland U. Haendeler J. Zeiher A. M. (1997). Angiotensin II induces apoptosis of human endothelial cells. Protective effect of nitric oxide. *Circ. Res.* 81 970–976. 10.1161/01.RES.81.6.970 9400377

[B19] Ewert S. Laesser M. Johansson B. Holm M. Aneman A. Fandriks L. (2003). The angiotensin II receptor type 2 agonist CGP 42112A stimulates NO production in the porcine jejunal mucosa. *BMC Pharmacol.* 3:2. 10.1186/1471-2210-3-2 12689346 PMC153509

[B20] Ferrario C. M. Strawn W. B. (2006). Role of the renin-angiotensin-aldosterone system and proinflammatory mediators in cardiovascular disease. *Am. J. Cardiol.* 98 121–128. 10.1016/j.amjcard.2006.01.059 16784934

[B21] Filipeanu C. M. Henning R. H. de Zeeuw D. Nelemans A. (2001). Intracellular angiotensin II and cell growth of vascular smooth muscle cells. *Br. J. Pharmacol.* 132 1590–1596. 10.1038/sj.bjp.0703984 11264254 PMC1572710

[B22] Fouda A. Y. Pillai B. Dhandapani K. M. Ergul A. Fagan S. C. (2017). Role of interleukin-10 in the neuroprotective effect of the angiotensin type 2 receptor agonist, compound 21, after ischemia/reperfusion injury. *Eur. J. Pharmacol.* 799 128–134. 10.1016/j.ejphar.2017.02.016 28192099 PMC5411859

[B23] Gao J. Zucker I. H. Gao L. (2014). Activation of central angiotensin type 2 receptors by compound 21 improves arterial baroreflex sensitivity in rats with heart failure. *Am. J. Hypertens.* 27 1248–1256. 10.1093/ajh/hpu044 24687998 PMC4229732

[B24] Gohlke P. Pees C. Unger T. (1998). AT2 receptor stimulation increases aortic cyclic GMP in SHRSP by a kinin-dependent mechanism. *Hypertension* 31(1 Pt 2) 349–355. 10.1161/01.HYP.31.1.349 9453327

[B25] Gul R. Ramdas M. Mandavia C. H. Sowers J. R. Pulakat L. (2012). RAS-mediated adaptive mechanisms in cardiovascular tissues: confounding factors of ras blockade therapy and alternative approaches. *Cardiorenal Med.* 2 268–280. 10.1159/000343456 23381810 PMC3551408

[B26] Gwathmey T. M. Alzayadneh E. M. Pendergrass K. D. Chappell M. C. (2012). Novel roles of nuclear angiotensin receptors and signaling mechanisms. *Am. J. Physiol. Regul. Integr. Comp. Physiol.* 302 R518–R530. 10.1152/ajpregu.00525.2011 22170620 PMC3311515

[B27] Harada S. Nakata T. Oguni A. Kido H. Hatta T. Fukuyama R. (2002). Contrasting effects of angiotensin type 1 and 2 receptors on nitric oxide release under pressure. *Hypertens. Res.* 25 779–786. 10.1291/hypres.25.779 12452333

[B28] Herst P. M. Berridge M. V. (2007). Cell surface oxygen consumption: a major contributor to cellular oxygen consumption in glycolytic cancer cell lines. *Biochim. Biophys. Acta* 1767 170–177. 10.1016/j.bbabio.2006.11.018 17266920

[B29] Inagami T. (1994). The renin-angiotensin system. *Essays Biochem.* 28 147–164. 7925317

[B30] Jensen P. K. (1966). Antimycin-insensitive oxidation of succinate and reduced nicotinamide-adenine dinucleotide in electron-transport particles. I. pH dependency and hydrogen peroxide formation. *Biochim. Biophys. Acta* 122 157–166. 10.1016/0926-6593(66)90057-9 4291041

[B31] Jones E. S. Vinh A. McCarthy C. A. Gaspari T. A. Widdop R. E. (2008). AT2 receptors: functional relevance in cardiovascular disease. *Pharmacol. Ther.* 120 292–316. 10.1016/j.pharmthera.2008.08.009 18804122 PMC7112668

[B32] Kambayashi Y. Bardhan S. Takahashi K. Tsuzuki S. Inui H. Hamakubo T. (1993). Molecular cloning of a novel angiotensin II receptor isoform involved in phosphotyrosine phosphatase inhibition. *J. Biol. Chem.* 268 24543–24546. 8227011

[B33] Khaddaj Mallat R. Mathew John C. Kendrick D. J. Braun A. P. (2017). The vascular endothelium: a regulator of arterial tone and interface for the immune system. *Crit. Rev. Clin. Lab. Sci.* 54 458–470. 10.1080/10408363.2017.1394267 29084470

[B34] Kijima K. Matsubara H. Murasawa S. Maruyama K. Mori Y. Inada M. (1995). Gene transcription of angiotensin II type 2 receptor is repressed by growth factors and glucocorticoids in PC12 cells. *Biochem. Biophys. Res. Commun.* 216 359–366. 10.1006/bbrc.1995.2632 7488113

[B35] Kim N. Jung Y. Nam M. Sun Kang M. Lee M. K. Cho Y. (2017). Angiotensin II affects inflammation mechanisms via AMPK-related signalling pathways in HL-1 atrial myocytes. *Sci. Rep.* 7:10328. 10.1038/s41598-017-09675-3 28871102 PMC5583339

[B36] Kim S. Y. Kim S. J. Kim B. J. Rah S. Y. Chung S. M. Im M. J. (2006). Doxorubicin-induced reactive oxygen species generation and intracellular Ca2+ increase are reciprocally modulated in rat cardiomyocytes. *Exp. Mol. Med.* 38 535–545. 10.1038/emm.2006.63 17079870

[B37] Kumar V. Knowle D. Gavini N. Pulakat L. (2002). Identification of the region of AT2 receptor needed for inhibition of the AT1 receptor-mediated inositol 1,4,5-triphosphate generation. *FEBS Lett.* 532 379–386. 10.1016/S0014-5793(02)03713-4 12482596

[B38] Kurisu S. Ozono R. Oshima T. Kambe M. Ishida T. Sugino H. (2003). Cardiac angiotensin II type 2 receptor activates the kinin/NO system and inhibits fibrosis. *Hypertension* 41 99–107. 10.1161/01.HYP.0000050101.90932.14 12511537

[B39] Li D. Y. Zhang Y. C. Philips M. I. Sawamura T. Mehta J. L. (1999). Upregulation of endothelial receptor for oxidized low-density lipoprotein (LOX-1) in cultured human coronary artery endothelial cells by angiotensin II type 1 receptor activation. *Circ. Res.* 84 1043–1049. 10.1161/01.RES.84.9.1043 10325241

[B40] Li H. P. Qiu H. B. Wang H. Q. (2015). Effect of lipopolysaccharide on angiotensin II type 1 receptor expression and function in human pulmonary microvascular endothelial cells. *Mol. Med. Rep.* 12 8289–8293. 10.3892/mmr.2015.4481 26497066

[B41] Li J. Culman J. Hortnagl H. Zhao Y. Gerova N. Timm M. (2005). Angiotensin AT2 receptor protects against cerebral ischemia-induced neuronal injury. *FASEB J.* 19 617–619. 10.1096/fj.04-2960fje 15665034

[B42] Li M. Tejada T. Lambert J. P. Nicholson C. K. Yahiro E. Ambai V. T. (2016). Angiotensin type 2-receptor (AT2R) activation induces hypotension in apolipoprotein E-deficient mice by activating peroxisome proliferator-activated receptor-gamma. *Am. J. Cardiovasc. Dis.* 6 118–128. 27679746 PMC5030391

[B43] Liu Y. H. Yang X. P. Sharov V. G. Nass O. Sabbah H. N. Peterson E. (1997). Effects of angiotensin-converting enzyme inhibitors and angiotensin II type 1 receptor antagonists in rats with heart failure. Role of kinins and angiotensin II type 2 receptors. *J. Clin. Invest.* 99 1926–1935. 10.1172/JCI119360 9109437 PMC508017

[B44] Ludwig M. Steinhoff G. Li J. (2012). The regenerative potential of angiotensin AT2 receptor in cardiac repair. *Can. J. Physiol. Pharmacol.* 90 287–293. 10.1139/y11-108 22364522

[B45] Lum-Naihe K. T. Toedebusch R. Mahmood A. Bajwa J. Carmack T. Kumar S. (2017). Cardiovascular disease progression in female Zucker Diabetic Fatty rats occurs via unique mechanisms compared to males. *Sci. Rep.* 7:17823. 10.1038/s41598-017-18003-8 29259233 PMC5736602

[B46] Mahmood A. Pulakat L. (2015). Differential effects of beta-blockers, angiotensin ii receptor blockers, and a novel AT2R agonist NP-6A4 on stress response of nutrient-starved cardiovascular cells. *PLoS One* 10:e0144824. 10.1371/journal.pone.0144824 26691397 PMC4686716

[B47] Masaki H. Kurihara T. Yamaki A. Inomata N. Nozawa Y. Mori Y. (1998). Cardiac-specific overexpression of angiotensin II AT2 receptor causes attenuated response to AT1 receptor-mediated pressor and chronotropic effects. *J. Clin. Invest.* 101 527–535. 10.1172/JCI1885 9449684 PMC508594

[B48] Matavelli L. C. Zatz R. Siragy H. M. (2015). A nonpeptide angiotensin II type 2 receptor agonist prevents renal inflammation in early diabetes. *J. Cardiovasc. Pharmacol.* 65 371–376. 10.1097/FJC.0000000000000207 25590749 PMC4390440

[B49] Matrougui K. Loufrani L. Heymes C. Levy B. I. Henrion D. (1999). Activation of AT(2) receptors by endogenous angiotensin II is involved in flow-induced dilation in rat resistance arteries. *Hypertension* 34(4 Pt 1) 659–665. 10.1161/01.HYP.34.4.659 10523343

[B50] McCarthy C. A. Vinh A. Miller A. A. Hallberg A. Alterman M. Callaway J. K. (2014). Direct angiotensin AT2 receptor stimulation using a novel AT2 receptor agonist, compound 21, evokes neuroprotection in conscious hypertensive rats. *PLoS One* 9:e95762. 10.1371/journal.pone.0095762 24752645 PMC3994132

[B51] Minotti G. Menna P. Salvatorelli E. Cairo G. Gianni L. (2004). Anthracyclines: molecular advances and pharmacologic developments in antitumor activity and cardiotoxicity. *Pharmacol. Rev.* 56 185–229. 10.1124/pr.56.2.6 15169927

[B52] Miyata N. Park F. Li X. F. Cowley A. W. Jr. (1999). Distribution of angiotensin AT1 and AT2 receptor subtypes in the rat kidney. *Am. J. Physiol.* 277(3 Pt 2) F437–F446. 10.1152/ajprenal.1999.277.3.F437 10484527

[B53] Moudgil R. Musat-Marcu S. Xu Y. Kumar D. Jugdutt B. I. (2002). Increased AT(2)R protein expression but not increased apoptosis during cardioprotection induced by AT(1)R blockade. *Can. J. Cardiol.* 18 1107–1116. 12420045

[B54] Mukoyama M. Nakajima M. Horiuchi M. Sasamura H. Pratt R. E. Dzau V. J. (1993). Expression cloning of type 2 angiotensin II receptor reveals a unique class of seven-transmembrane receptors. *J. Biol. Chem.* 268 24539–24542. 8227010

[B55] Nguyen Dinh Cat A. Montezano A. C. Burger D. Touyz R. M. (2013). Angiotensin II, NADPH oxidase, and redox signaling in the vasculature. *Antioxid. Redox Signal.* 19 1110–1120. 10.1089/ars.2012.4641 22530599 PMC3771549

[B56] Nora E. H. Munzenmaier D. H. Hansen-Smith F. M. Lombard J. H. Greene A. S. (1998). Localization of the ANG II type 2 receptor in the microcirculation of skeletal muscle. *Am. J. Physiol.* 275(4 Pt 2) H1395–H1403. 10.1152/ajpheart.1998.275.4.H1395 9746490

[B57] Oishi Y. Ozono R. Yoshizumi M. Akishita M. Horiuchi M. Oshima T. (2006). AT2 receptor mediates the cardioprotective effects of AT1 receptor antagonist in post-myocardial infarction remodeling. *Life Sci.* 80 82–88. 10.1016/j.lfs.2006.08.033 17023005

[B58] Okumura M. Iwai M. Ide A. Mogi M. Ito M. Horiuchi M. (2005). Sex difference in vascular injury and the vasoprotective effect of valsartan are related to differential AT2 receptor expression. *Hypertension* 46 577–583. 10.1161/01.HYP.0000178564.14464.80 16103268

[B59] Padia S. H. Carey R. M. (2013). AT2 receptors: beneficial counter-regulatory role in cardiovascular and renal function. *Pflugers Arch.* 465 99–110. 10.1007/s00424-012-1146-3 22949090 PMC3548020

[B60] Pandey A. Gaikwad A. B. (2017). Compound 21 and telmisartan combination mitigates type 2 diabetic nephropathy through amelioration of caspase mediated apoptosis. *Biochem. Biophys. Res. Commun.* 487 827–833. 10.1016/j.bbrc.2017.04.134 28456626

[B61] Peluso A. A. Bertelsen J. B. Andersen K. Mortsensen T. P. Hansen P. B. Sumners C. (2018). Identification of protein phosphatase involvement in the AT2 receptor-induced activation of endothelial nitric oxide synthase. *Clin. Sci.* 132 777–790. 10.1042/CS20171598 29540539

[B62] Prieto D. Contreras C. Sanchez A. (2014). Endothelial dysfunction, obesity and insulin resistance. *Curr. Vasc. Pharmacol.* 12 412–426. 10.2174/1570161112666140423221008 24846231

[B63] Re R. (2007). Intracellular renin-angiotensin system: the tip of the intracrine physiology iceberg. *Am. J. Physiol. Heart Circ. Physiol.* 293 H905–H906. 10.1152/ajpheart.00552.2007 17526648

[B64] Rehman A. Leibowitz A. Yamamoto N. Rautureau Y. Paradis P. Schiffrin E. L. (2012). Angiotensin type 2 receptor agonist compound 21 reduces vascular injury and myocardial fibrosis in stroke-prone spontaneously hypertensive rats. *Hypertension* 59 291–299. 10.1161/HYPERTENSIONAHA.111.180158 22184324

[B65] Sadoshima J. Izumo S. (1993). Molecular characterization of angiotensin II–induced hypertrophy of cardiac myocytes and hyperplasia of cardiac fibroblasts. Critical role of the AT1 receptor subtype. *Circ. Res.* 73 413–423. 10.1161/01.RES.73.3.413 8348686

[B66] Sampson A. K. Irvine J. C. Shihata W. A. Dragoljevic D. Lumsden N. Huet O. (2016). Compound 21, a selective agonist of angiotensin AT2 receptors, prevents endothelial inflammation and leukocyte adhesion *in vitro* and *in vivo*. *Br. J. Pharmacol.* 173 729–740. 10.1111/bph.13063 25560767 PMC4742292

[B67] Sampson A. K. Moritz K. M. Jones E. S. Flower R. L. Widdop R. E. Denton K. M. (2008). Enhanced angiotensin II type 2 receptor mechanisms mediate decreases in arterial pressure attributable to chronic low-dose angiotensin II in female rats. *Hypertension* 52 666–671. 10.1161/HYPERTENSIONAHA.108.114058 18711010

[B68] Sonowal H. Pal P. Shukla K. Saxena A. Srivastava S. K. Ramana K. V. (2018). Aldose reductase inhibitor, fidarestat prevents doxorubicin-induced endothelial cell death and dysfunction. *Biochem. Pharmacol.* 150 181–190. 10.1016/j.bcp.2018.02.018 29458045 PMC5866779

[B69] Wang Z. Q. Moore A. F. Ozono R. Siragy H. M. Carey R. M. (1998). Immunolocalization of subtype 2 angiotensin II (AT2) receptor protein in rat heart. *Hypertension* 32 78–83. 10.1161/01.HYP.32.1.78 9674641

[B70] Widdop R. E. Jones E. S. Hannan R. E. Gaspari T. A. (2003). Angiotensin AT2 receptors: cardiovascular hope or hype? *Br. J. Pharmacol.* 140 809–824. 10.1038/sj.bjp.0705448 14530223 PMC1574085

[B71] World Health Organization [WHO] (2017). *Cardiovascular Diseases (CVD)*. Available at: http://www.who.int/mediacentre/factsheets/fs317/en/

[B72] Wu L. Iwai M. Nakagami H. Chen R. Suzuki J. Akishita M. (2002). Effect of angiotensin II type 1 receptor blockade on cardiac remodeling in angiotensin II type 2 receptor null mice. *Arterioscler. Thromb. Vasc. Biol.* 22 49–54. 10.1161/hq0102.102277 11788460

[B73] Xu J. Sun Y. Carretero O. A. Zhu L. Harding P. Shesely E. G. (2014). Effects of cardiac overexpression of the angiotensin II type 2 receptor on remodeling and dysfunction in mice post-myocardial infarction. *Hypertension* 63 1251–1259. 10.1161/HYPERTENSIONAHA.114.03247 24732892 PMC4031246

[B74] Yang J. Chen C. Ren H. Han Y. He D. Zhou L. (2012). Angiotensin II AT(2) receptor decreases AT(1) receptor expression and function via nitric oxide/cGMP/Sp1 in renal proximal tubule cells from Wistar-Kyoto rats. *J. Hypertens.* 30 1176–1184. 10.1097/HJH.0b013e3283532099 22504846 PMC3705562

[B75] Yayama K. Okamoto H. (2008). Angiotensin II-induced vasodilation via type 2 receptor: role of bradykinin and nitric oxide. *Int. Immunopharmacol.* 8 312–318. 10.1016/j.intimp.2007.06.012 18182246

[B76] Zhu L. Carretero O. A. Liao T. D. Harding P. Li H. Sumners C. (2010). Role of prolylcarboxypeptidase in angiotensin II type 2 receptor-mediated bradykinin release in mouse coronary artery endothelial cells. *Hypertension* 56 384–390. 10.1161/HYPERTENSIONAHA.110.155051 20606103 PMC2924447

