# Supplementary figures and images for: Cell-Specific Protective Signaling Induced by the Novel AT2R-Agonist NP-6A4 on Human Endothelial and Smooth Muscle Cells
Source: Front Pharmacol. 2018 Aug 21;9:928. doi: 10.3389/fphar.2018.00928 (PMC6111462; doi:10.3389/fphar.2018.00928)

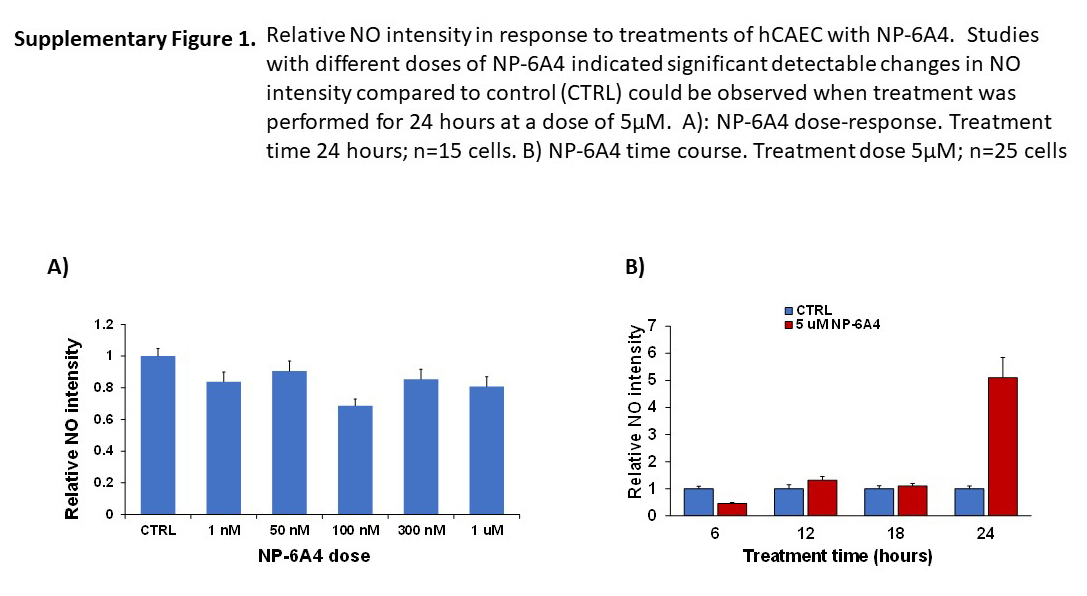

Supplement: Supplementary file 1 [file Image_1.JPEG]

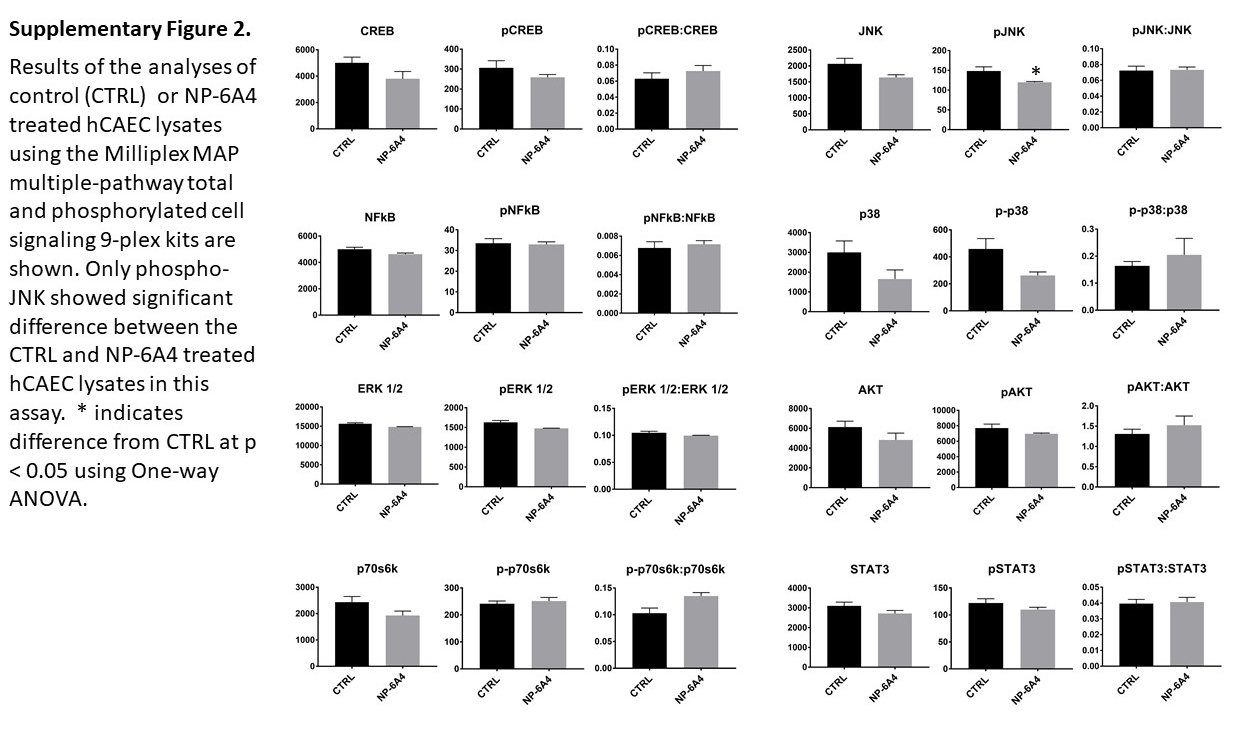

Supplement: Supplementary file 2 [file Image_2.JPEG]
